# Distribution, diversity patterns and faunogenesis of the millipedes (Diplopoda) of the Himalayas

**DOI:** 10.3897/zookeys.741.20041

**Published:** 2018-03-07

**Authors:** Sergei I. Golovatch, Jochen Martens

**Affiliations:** 1 Institute for Problems of Ecology and Evolution, Russian Academy of Sciences, Leninsky prospekt 33, Moscow 119071 Russia; 2 Institute of Organismic and Molecular Evolutionary Biology, Johannes Gutenberg University of Mainz, D-55099 Mainz, Germany

**Keywords:** Diplopoda, faunistics, Plio-Pleistocene, Asia, Palaearctic

## Abstract

The Himalayas support a highly rich, diverse, multi-layered, mostly endemic diplopod fauna which presently contains >270 species, 53 genera, 23 families and 13 orders. This is the result of mixing the ancient, apparently Tertiary and younger, Plio-Pleistocene elements of various origins, as well as the most recent anthropochore (= man-mediated) introductions. At the species and, partly, generic levels, the fauna is largely autochthonous and sylvicolous, formed through abounding *in situ* radiation and vicariance events. In general, the species from large genera and families tend to occupy a wide range of altitudes, but nearly each of the constituent species shows a distribution highly localized both horizontally and altitudinally, yet quite often with sympatry or even syntopy involved. The bulk of the fauna is Indo-Malayan in origin, with individual genera or families shared with those of SE Asia (mostly) and/or S India (few). Sino-Himalayan and, especially, Palaearctic components are subordinate, but also clearly distinguishable.

## Introduction

The Himalaya Range, or Himalayas for short, meaning “the abode of snow” in Sanskrit, is the mountain range in Asia that separates the Indian subcontinent from the Tibetan Plateau. Sometimes by extension, it is also the name of a massive mountain system that includes the Karakoram, the Hindu Kush, and other, lesser, ranges that reach out from the Pamir Knot (http://maps.thefullwiki.org/Himalayas). However, below the Himalayas is treated in the strict sense. The main Himalayan Range runs, northwest to southeast, from the Indus River valley to the Brahmaputra River valley, forming an arc which varies in width from 400 km in the western Kashmir-Xinjiang region to 150 km in the southeastern Tibet-Arunachal Pradesh region. The range consists of three extensive sub-ranges, with the northernmost, and highest, known as the Great Himalayas.

**Figure 1.**
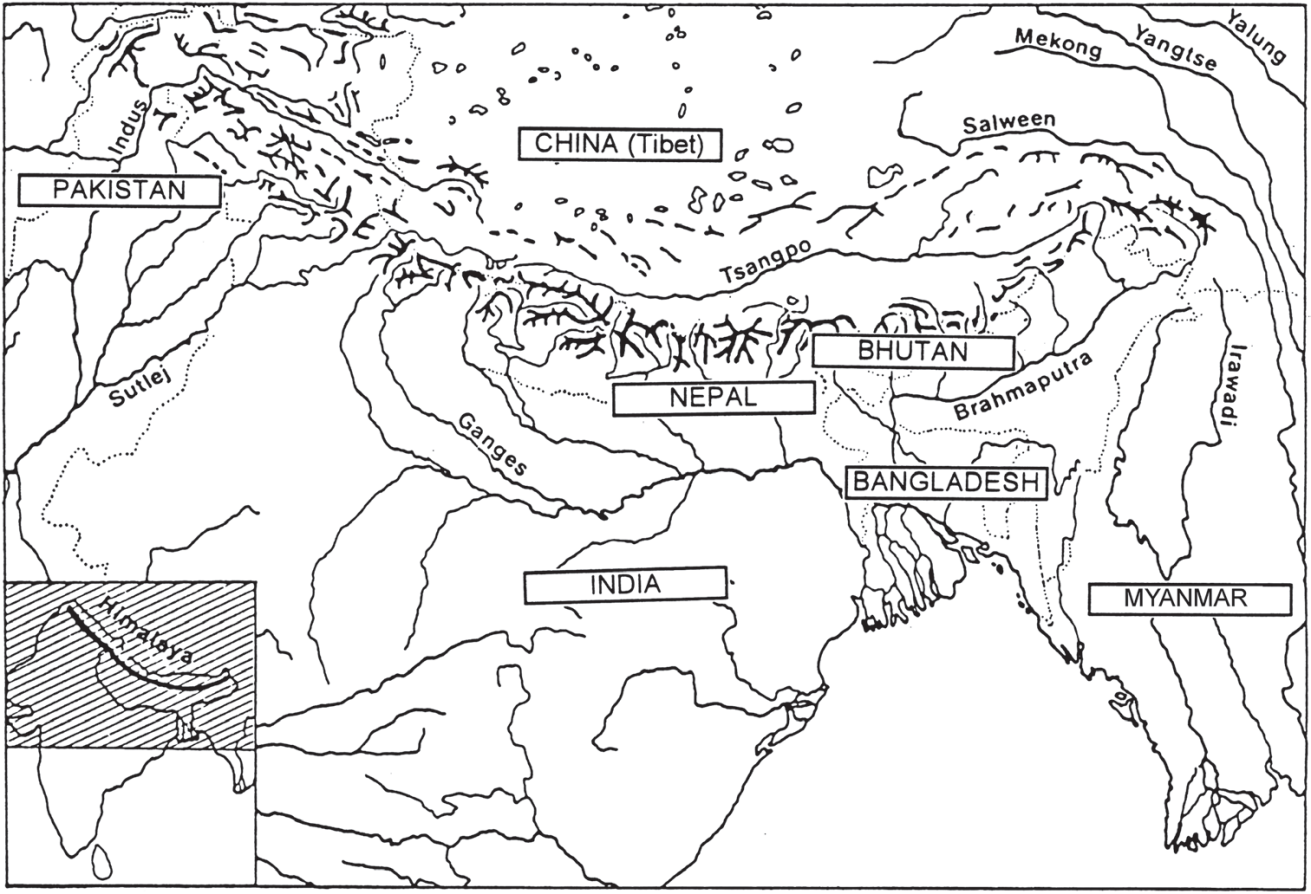
Orographic map of the Himalayan region.

The Himalayan mountain system is the Earth’s highest and home to the world’s highest peaks, the Eight-thousanders, which include Mount Everest and K2. The system, which includes various outlying sub-ranges, stretches across five countries: India, Nepal, Bhutan, China and Pakistan. The Himalayan Range is bordered on the northwest by the Karakoram and Hindu Kush ranges, on the north by the Tibetan Plateau, and on the south by the Indo-Gangetic Plain. The region is roughly delimited by 74°E in the west and 95°E in the east. Some of the world’s major rivers, the Indus, the Ganges, and the Tsangpo-Brahmaputra, rise in the Himalayas, and their combined drainage basin is home to some 600 million people. The Himalayas have profoundly shaped the cultures of South Asia, having united and separated them as well; many Himalayan peaks are sacred in Hinduism and Buddhism (https://en.wikipedia.org/wiki/Himalayas). An orographic map of the Himalayas is presented in Fig. 1.

From a biologist’s viewpoint, the Himalayas represent a highly important biogeographical barrier between the cold and arid uplands of Central Asia and the largely tropical South and Southeast Asia. During the southwestern monsoon period, precipitation mainly occurs on the southern slopes, being greatly reduced on the northern ones. This picture is especially typical of the Central Himalayas, more or less within Kumaon, Nepal, Sikkim and Bhutan, as more to the west the aridity of Central Asia extends across the southern slopes while in the eastern parts of the system heavy monsoon rains, though declining in amount and frequency, reach beyond the main ridge as far as southeastern Tibet (Troll 1967, Miehe 2015).

**Figure 2.**
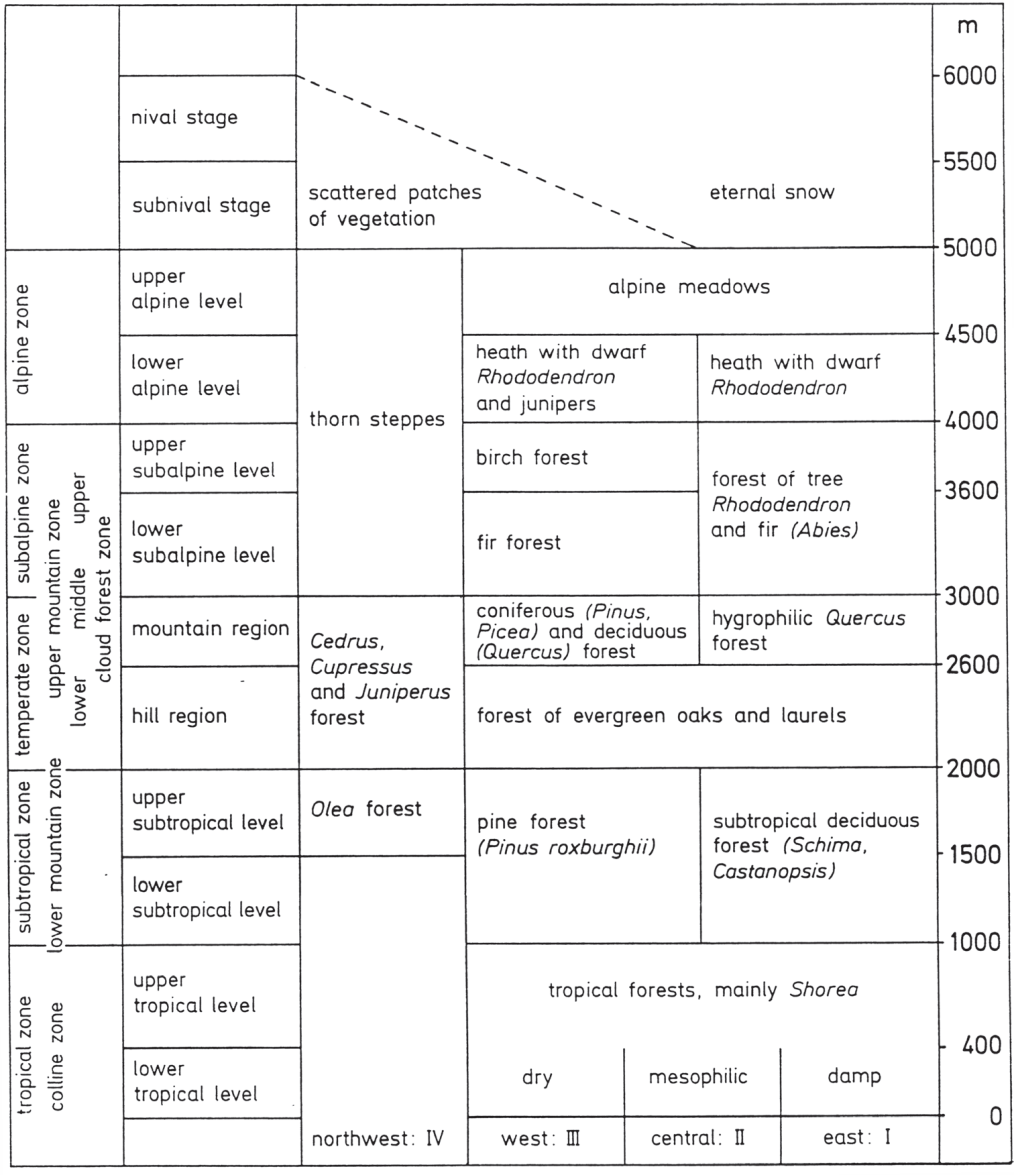
The vegetation belts and most important plant communities in the Nepal Himalayas. The Roman numerals at the bottom indicate the floral regions of Nepal (modified, after Dobremez 1972).

The drastic climatic gradient within the Central Himalayas is of utmost importance in affecting the distribution of various organisms. Although phyto- and zoogeographical regions delimited differ to some degree, they both emphasize the role of the Himalayas as a contact zone between two major biogeographical realms, the Palaearctic and Oriental, which meet and intermesh here in various combinations. All areas lying north of the Central Himalayas obviously belong to the Palaearctic, as do the highest parts of the inhabited southern macroslope. The lower and lowest elevations of the southern macroslope are largely attributable to the Oriental, or Indo-Malayan realm. In addition, a third realm, the Sino-Himalayan biogeographical region, can be distinguished, bringing old faunal elements into the Himalayan chain. However, the border between both regions is generally neither striking nor abrupt, forming more (especially in the eastern Himalayas) or less (in their central parts) vast transition areas, numerous inversions or anomalies. In other words, the otherwise manifest rule “(sub)tropical organisms for (sub)tropical environments only” is very often violated in the Himalayas, particularly in the central parts of the system and as regards animals in general (Martens 1984, 1993, 2015). Even the pattern of vertical zonation of the tree plant cover in the region is rather conventional (Dobremez 1972) (Fig. 2).

The first, provisional review of the millipede fauna of the Himalayas (Golovatch and Martens 1996) was based on a fauna of approximately 200 species or subspecies. Now, the list has reached more than 270 species or subspecies belonging to 53 genera distributed in 23 families and 13 orders (Table 1). As before, several species and even genera remain unidentified. The main increase is due to the omnipresent family Paradoxosomatidae, especially as regards the fauna of Nepal. The objective of this paper is to critically list the actually known Himalayan diplopod fauna and to discuss the different faunal and evolutionary influences that made this fauna so rich and complex.

## Material and methods

These results mostly rely on published records, which have grown considerably over the past two decades. The resultant checklist (Table 1) is not just a literature compilation, as it is largely based on the vast experience of the first author who has described numerous diplopod species from the Himalayas. Most of the recent advance has appeared, based on material collected by the second author and/or his collaborators during numerous, often long-term expeditions to Nepal, India and elsewhere. The trips to Nepal covered all seasons, focused on the exploration of local forest soil/litter fauna and concerned all forest biomes ranging from the terai lowlands to and beyond the timber line.

**Table 1.** Diplopoda of the Himalayas. In addition to the taxonomic composition of the fauna, data on the vertical and geographical distribution of species in the region are also presented, largely with key references.

Fauna	Altitude (m a.s.l.)	Country/state and main reference(s)
**Order Polyxenida**	1585–2400	
**Family Polyxenidae**	1585–2400	
Genus *Polyxenus* Latreille, 1802–03		
1. *Polyxenus* sp.	1585	India, Jammu & Kashmir (Silvestri 1936)
Genus *Monographis* Attems, 1907		
2. *Monographis mira* (Turk, 1947)	1600–2400	Nepal^1^ & India, Almora (Turk 1947, Condé 1962, Golovatch and Wesener 2016)
Genus *Unixenus* Jones, 1944		
3. *Unixenus* sp.	2400–4550	Nepal^2^ (Condé and Jacquemin-Nguyen Duy 1968, Golovatch and Martens 1996)
**Order Sphaerotheriida**	140–2700	
**Family Zephroniidae**	140–2700	
Genus *Indosphaera* Attems, 1935		
4. *Indosphaera feae* Attems, 1935	?	India, Assam (Attems 1936, Golovatch and Wesener 2016)
Genus *Kophosphaera* Attems, 1935	1650–2100	
5. *Kophosphaera brevilamina* Attems, 1936	1700	India, West Bengal & Darjeeling Distr. (Golovatch and Martens 1996, Golovatch and Wesener 2016)
6. *K. devolvens* Attems, 1936	1700–2050	India, Sikkim & Darjeeling Distr. (Golovatch and Martens 1996, Golovatch and Wesener 2016)
7. *K. excavata* (Butler, 1874)	?	Nepal, Chitlang; India, Sikkim, Darjeeling Distr. & Assam (Golovatch and Martens 1996, Golovatch and Wesener 2016)
8. *K. mammifera* Attems, 1936	?	India, Darjeeling Distr. & Assam (Golovatch and Martens 1996, Golovatch and Wesener 2016)
9. *K. martensi* Wesener, 2015	2100	Nepal (Wesener 2015)
10. *K. politissima* Attems, 1935	1650–1870	India, Darjeeling Distr. & Nepal (Wesener 2015)
11. *K. shivapuri* Wesener, 2015	1700–2100	Nepal (Wesener 2015)
Genus *Zephronia* Gray, 1832	140–2700	
12. *Zephronia alticola alticola* Attems, 1936	400–1700	India, Darjeeling Distr. & Assam (Attems 1936, Golovatch and Martens 1996)
13. *Z. alticola bengalica* Attems, 1936	?	India, West Bengal (Attems 1936, Golovatch and Martens 1996)
14. *Z. debilis* Attems, 1936	1700	India, Darjeeling Distr. (Attems 1936, Golovatch and Martens 1996)
15. *Z. densipora* Attems, 1936	?	India, Assam (Attems 1936, Golovatch and Martens 1996)
16. *Z. disparipora* Attems, 1936	140	India, Assam (Attems 1936, Golovatch and Martens 1996)
17. *Z. hirta* Attems, 1936	1700	India, Darjeeling Distr. (Attems 1936, Golovatch and Martens 1996)
18. *Z. hysophila* Attems, 1936	?	India, Assam (Attems 1936, Golovatch and Martens 1996)
19. *Z. juvenis* Attems, 1936	?	India, Assam (Attems 1936, Golovatch and Martens 1996)
20. *Z. lignivora* Attems, 1936	180–330	India, Assam (Attems 1936, Golovatch and Martens 1996)
21. *Z. manca* Attems, 1936	1000–1700	Vietnam & India, Darjeeling Distr. (Attems 1936, Golovatch and Martens 1996)
22. *Z. montana* (Karsch, 1881)	?	“Himalaya” (Wesener 2015)
23. *Z. nepalensis* Wesener, 2015	1700–2600	Nepal (Wesener 2015)
24. *Z. nigrinota* Butler, 1872	2300–2700	India, Darjeeling Distr. (Golovatch and Martens 1996, Golovatch and Wesener 2016)
25. *Z. specularis* Attems, 1936	?	India, Assam (Attems 1936, Golovatch and Martens 1996)
26. *Z. tigrina* Butler, 1872	?	India, Darjeeling Distr. (Golovatch and Wesener 2016)
27. *Z. tigrinoides* Attems, 1936	170	India, Darjeeling Distr. (Attems 1936, Golovatch and Martens 1996)
28. *Z. tumida* Butler, 1882	?	India, Assam & Darjeeling Distr.; Myanmar (Wesener 2015)
**Order Glomerida**	150–3300	
**Family Glomeridae**	150–3300	
Genus *Hyleoglomeris* Verhoeff, 1910	150–3300	
29. *Hyleoglomeris crassipes* Golovatch, 1987	2450–2720	Nepal (Golovatch 1987b, Golovatch and Martens 1996)
30. *H. electa* (Silvestri, 1917)	500–1700	India, Darjeeling Distr. (Attems 1936, Golovatch and Martens 1996)
31. *H. gorkhalis* Golovatch, 1987	1200	Nepal (Golovatch 1987b, Golovatch and Martens 1996)
32. *H. khumbua* Golovatch, 1987	3250–3300	Nepal (Golovatch 1987b, Golovatch and Martens 1996)
33. *H. modesta* Silvestri, 1917	150	India, Assam (Golovatch and Martens 1996)
34. *H. nagarjunga* Golovatch, 1987	1600–2100	Nepal (Golovatch 1987b, Golovatch and Martens 1996, Golovatch et al. 2006)
35. *H. tinjurana* Golovatch, 1987	2450	Nepal (Golovatch 1987b, Golovatch and Martens 1996)
36. *H. venustula* Silvestri, 1917	?	India, Assam (Golovatch and Martens 1996)
**Order Siphonophorida**		
**Family Siphonorhinidae**	500–1700	
Genus *Siphonorhinus* Pocock, 1894	500–1700	
37. *Siphonorhinus cingulatus* (Attems, 1936)	500–1700	Vietnam and India, Darjeeling Distr. (Attems 1936, Golovatch and Wesener 2016)
38. *S. coniceps* (Attems, 1936)	1700	India, Darjeeling Distr. (Attems 1936, Golovatch and Wesener 2016)
39. *S. larwoodi* (Turk, 1947)	1600	India, Almora (Golovatch and Martens 1996, Golovatch and Wesener 2016)
**Order Siphonocryptida**		
**Family Siphonocryptidae**	2450	
Genus *Hirudicryprus* Enghoff & Golovatch, 1995		
40. *Hirudicryprus quintumelementum* Korsós, Geoffroy & Mauriès, 2009	2450	Nepal (Korsós et al. 2009)
**Order Platydesmida**	<2000	
**Family Andrognathidae**	<2000	
Genus *Pseudodesmus* Pocock, 1887		
41. ?*Pseudodesmus* sp.	<2000	Nepal (Golovatch and Martens 1996)
**Order Polyzoniida**	4700–4800	
**Family Hirudisomatidae**	4700–4800	
Genus *Nepalozonium* Shelley, 1996		
42. *Nepalozonium trimaculatum* Shelley, 1996	4700–4800	Nepal (Shelley 1996)
**Order Chordeumatida**	900–4100	
**Family Kashmireumatidae**	2600–4100	
Genus *Kashmireuma* Mauriès, 1982	2600–4100	
43. *Kashmireuma nepalensis* Mauriès, 1988	3600–4100	Nepal (Mauriès 1988, Golovatch and Martens 1996)
44. *K. nielseni* Mauriès, 1982	2600–3500	India, Kashmir (Mauriès 1982, Golovatch and Martens 1996)
45. *K. schawalleri* Shear, 1987	3450–3600	Nepal (Shear 1987, Golovatch and Martens 1996)
**Family Cleidogonidae**	900–3900	
Genus *Tianella* Attems, 1904	900–3900	
46. *Tianella ausobskyi* Shear, 1987	2500–3050	Nepal (Shear 1987, Golovatch and Martens 1996)
47. *T. bobanga* Shear, 1979	2460–2500	Nepal (Shear 1979, 1987, Golovatch and Martens 1996)
48. *T. daamsae* Shear, 1987	3600–3900	Nepal (Shear 1987, Golovatch and Martens 1996)
49. *T. gitanga* Shear, 1987	2550	Nepal (Shear 1987, Golovatch and Martens 1996)
50. *T. jaljalensis* Mauriès, 1988	2350	Nepal (Mauriès 1988, Golovatch and Martens 1996)
51. *T. kathmandua* Mauriès, 1988	1700	Nepal (Mauriès 1988, Golovatch and Martens 1996)
52. *T. lughla* Shear, 1979	2950–3300	Nepal (Shear 1979, 1987, Golovatch and Martens 1996)
53. *T. managa* Shear, 1987	2550	Nepal (Shear 1987, Golovatch and Martens 1996)
54. *T. mangsingma* Mauriès, 1988	2250	Nepal (Mauriès 1988, Golovatch and Martens 1996)
55. *T. martensi* Shear, 1979	1150–2900	Nepal (Shear, 1979, 1987, Golovatch and Martens 1996)
56. *T. smetanai* Mauriès, 1988	3250	Nepal (Mauriès 1988, Golovatch and Martens 1996)
57. *Tianella* sp.	900–1400	India, Darjeeling Distr. (Golovatch and Martens 1996)
**Family Megalotylidae**	1900–4100	
Genus *Nepalella* Shear, 1979	1900–4100	
58. *Nepalella deharvengi* Mauriès, 1988	2900–3500	Nepal (Mauriès 1988, Golovatch and Martens 1996)
59. *N. gairiensis* Mauriès, 1988	3000	Nepal (Mauriès 1988, Golovatch and Martens 1996)
60. *N. gunsa* Shear, 1987	3600–3800	Nepal (Shear 1987, Golovatch and Martens 1996)
61. *N. jaljalae* Mauriès, 1988	2200	Nepal (Mauriès 1988, Golovatch and Martens 1996)
62. *N. khumbua* Shear, 1979	3350–3300	Nepal (Shear 1979, 1987, Golovatch and Martens 1996)
63. *N. phulcokia* Mauriès, 1988	2250	Nepal (Mauriès 1988, Golovatch and Martens 1996)
64. *N. ringmoensis* Mauriès, 1988	2750–3000	Nepal (Mauriès 1988, Golovatch and Martens 1996)
65. *N. taplejunga* Shear, 1987	3000–3300	Nepal (Shear 1987, Golovatch and Martens 1996)
66. *N. thodunga* Shear, 1979	3200	Nepal (Shear 1979, 1987, Golovatch and Martens 1996)
67. *N. tragsindola* Mauriès, 1988	2450–3300	Nepal (Mauriès 1988, Golovatch and Martens 1996)
68. *Nepalella* sp.	1900–4100	Nepal (Golovatch and Martens 1996)
**Order Callipodida**	650	
**Family Caspiopetalidae**	650	
Genus *Bollmania* Silvestri, 1896		
69. *Bollmania kohalana* (Attems, 1936)	650	Pakistan, Punjab (Attems 1936, Golovatch and Wesener 2016)
**Order Julida**	1680–4800	
**Family Julidae**	1680–4800	
Genus *Anaulaciulus* Pocock, 1895	1900–4500	
70. *Anaulaciulus acaudatus* Korsós, 1996	3990	India, Sikkim (Korsós 1996, Golovatch and Martens 1996)
71. *A. bilineatus* Korsós, 1996	3300–4300	Nepal (Korsós 1996, Golovatch and Martens 1996)
72. *A. kashmirensis* Korsós, 1996	3100–3200	India, Kashmir (Korsós 1996, Golovatch and Martens 1996)
73. *A. nepalensis* Korsós, 1996	2600–3400	Nepal (Korsós 1996, Golovatch and Martens 1996)
74. *A. niger* Korsós, 1996	2600–4500	Nepal (Korsós 1996, Golovatch and Martens 1996)
75. *A. tibetanus* Korsós, 1996	3700	China, Tibet; India, Assam (Korsós 1996, Golovatch and Martens 1996)
76. *A. topali* Korsós, 1996	2300	India, Kashmir (Korsós 1996, Golovatch and Martens 1996)
Genus *Nepalmatoiulus* Mauriès, 1983	1680–4800	
77. *Nepalmatoiulus appendiculatus* Enghoff, 1987	1900–2100	India, Uttar Pradesh (Enghoff 1987, Golovatch and Martens 1996)
78. *N. deharvengi* (Mauriès, 1983)	2550–3350	Nepal (Mauriès 1983, Golovatch and Martens 1996)
79. *N. dhaulagiri* Enghoff, 1987	3000–3350	Nepal (Enghoff 1987, Golovatch and Martens 1996)
80. *N. generalis* Enghoff, 1987	3400	Nepal (Enghoff 1987, Golovatch and Martens 1996)
81. *N. hyalilobus* Enghoff, 1987	3600–3800	Nepal (Enghoff 1987, Golovatch and Martens 1996)
82. *N. ivanloebli* Enghoff, 1987	2200–4800	Nepal (Mauriès 1983, Enghoff 1987, Golovatch and Martens 1996)
83. *N. juctapositus* Enghoff, 1987	2800–3050	Nepal (Mauriès 1983, Golovatch and Martens 1996)
84. *N. martensi* Enghoff, 1987	3250–3300	Nepal (Enghoff 1987, Golovatch and Martens 1996)
85. *N. mauriesi* Enghoff, 1987	3600	Nepal (Enghoff 1983, Golovatch and Martens 1996)
86. *N. nigrescens* Enghoff, 1987	2300	Bhutan (Enghoff 1983, Golovatch and Martens 1996)
87. *N. pineti* Enghoff, 1987	2900	Nepal (Enghoff 1987, Golovatch and Martens 1996)
88. *N. rugiflagrum* Enghoff, 1987	3300	Bhutan (Enghoff 1987, Golovatch and Martens 1996)
89. *N. smetanai* (Mauriès, 1983)	1900–2700	Nepal (Enghoff 1983, Golovatch and Martens 1996)
90. *N. sympatricus* Enghoff, 1987	3000	Nepal (Enghoff 1987, Golovatch and Martens 1996)
88. *N. uncus* Enghoff, 1987	2550	Nepal (Enghoff 1987, Golovatch and Martens 1996)
91. *N. wuermlii* Enghoff, 1987	1680–2600	Bhutan (Enghoff 1987, Golovatch and Martens 1996)
92. *N. zachonoides* Enghoff, 1987	2450–2600	Nepal (Enghoff 1987, Golovatch and Martens 1996)
**Order Spirostreptida**	200–2500	
**Family Cambalopsidae**	<1000–1200	
Genus *Podoglyphiulus* Attems, 1909	<1000–1200	
93. *Podoglyphiulus elegans nepalensis* Mauriès, 1983	<1000	Nepal^3^ (Mauriès 1983, Golovatch and Martens 1996)
Genus *Trachyjulus* Peters, 1864		
94. *Trachyjulus mimus* Silvestri, 1924	1200	India, Assam (Silvestri 1924, Golovatch and Martens 1996, Golovatch and Wesener 2016)
95. *T. wilsonae* Mauriès, 1983	<1000	Nepal (Mauriès 1983, Golovatch and Martens 1996)
**Family Harpagophoridae**	200–2500	
Genus *Dametus* Attems, 1942		
96. *Dametus falcatus* (Attems, 1936)	400–500	India, Assam (Attems 1936, Golovatch and Wesener 2016)
Genus *Gonoplectus* Chamberlin, 1921	200–2500	
97. *Gonoplectus alius* Demange, 1961	?	India, Assam (Demange 1961, Golovatch and Martens 1996)
98. *G. bhutanensis* Demange, 1988	350–450	Bhutan (Demange 1988, Golovatch and Martens 1996)
99. *G. broelemanni* Demange, 1961	1800–2300	Nepal (Demange 1961, Golovatch and Martens 1996)
100. *G. corniger* (Attems, 1936)	?	India, Assam (Attems 1936, Golovatch and Martens 1996)
101. *G. gracilis* (Attems, 1936)	1200	India, Darjeeling Distr. (Attems 1936, Golovatch and Martens 1996)
102. *G. hyatti* Demange, 1961	1200	Nepal (Demange 1961, Golovatch and Martens 1996)
103. *G. malayus malayus* (Carl, 1909)	200–2500	Indonesia, Java; Nepal, Bhutan & India, Madhya Pradesh, Uttar Pradesh, Himachal Pradesh, West Bengal (Golovatch and Martens 1996, Golovatch and Wesener 2016)
104. *G. malayus lindbergi* (Carl, 1909)	350	Bhutan & India, Darjeeling Distr. (Golovatch and Martens 1996, Golovatch and Wesener 2016)
105. *G. probus* (Attems, 1936)	1000	India, Darjeeling Distr. (Attems 1936, Golovatch and Martens 1996, Golovatch and Wesener 2016)
106. *G. remyi* Demange, 1961	?	India, Assam (Demange 1961, Golovatch and Martens 1996, Golovatch and Wesener 2016)
107. *G. repertus* (Attems, 1936)	900	India, Darjeeling Distr. (Attems 1936, Golovatch and Wesener 2016)
108. *G. sulcatus* (Attems, 1936)	2400	India, Darjeeling Distr. (Attems 1936, Golovatch and Wesener 2016)
**Order Spirobolida**	<1000–1800	
**Family Pachybolidae**		
Genus *Trigoniulus* Pocock, 1894	<1000	
109. *Trigoniulus corallinus* (Gervais, 1847)	<1000	Pantropical, in India nearly throughout, including Assam (Golovatch and Wesener 2016)
**Family Pseudospirobolellidae**		
Genus *Physobolus* Attems, 1936		
110. *Physobolus olivaceus* Attems, 1936	1800	India, Darjeeling Distr. (Attems 1936, Golovatch and Wesener 2016)
**Order Polydesmida**	150–4500	
**Family Cryptodesmidae**		
Genus *Trichopeltis* Pocock, 1894	350–1000	
111. *Trichopeltis watsoni* Pocock, 1895	350–1000	Bangladesh, Myanmar, Bhutan and India, Darjeeling Distr., West Bengal, Assam & near Kolkata (Attems 1936, Golovatch and Martens 1996, Golovatch and Wesener 2016)
**Family Haplodesmidae**	150–1750	
Genus *Koponenius* Golovatch & VandenSpiegel, 2014	150–1750	
112. *Koponenius biramus* Golovatch & VandenSpiegel, 2014	1750	Nepal (Golovatch and VandenSpiegel 2014)
113. *K. schawalleri* Golovatch & VandenSpiegel, 2016	150	Nepal (Golovatch and VandenSpiegel 2016)
114. *K. unicornis* Golovatch & VandenSpiegel, 2014	880	India, Darjeeling Distr. (Golovatch and VandenSpiegel 2014, Golovatch and Wesener 2016)
**Family Opisotretidae**	1100–2440	
Genus *Martensodesmus* Golovatch, 1987	1100–2440	
115. *Martensodesmus bicuspidatus* Golovatch, 1988	1650–2000	Bhutan (Golovatch 1988a, Golovatch and Martens 1996, Golovatch et al. 2013)
116. *M. excornis* Golovatch, 1988	2440	Bhutan (Golovatch 1988a, Golovatch and Martens 1996, Golovatch et al. 2013)
117. *M. himalayensis* Golovatch, 1987	1100–1300	Nepal (Golovatch 1987a, Golovatch and Martens 1996, Golovatch et al. 2013)
118. *M. nagarjungicus* Golovatch, 1987	1900–2100	Nepal (Golovatch 1987a, Golovatch and Martens 1996, Golovatch et al. 2013)
119. *M. sherpa* Golovatch, 1987	1200	Nepal (Golovatch 1987a, Golovatch and Martens 1996, Golovatch et al. 2013)
120. *Martensodesmus* sp.	1300–2150	Nepal, Bhutan (Golovatch and Martens 1996)
**Family Paradoxosomatidae**	150–4500	
Genus *Anoplodesmus* Carl, 1932	1000–3600	
121. *Anoplodesmus affinis* (Golovatch, 1990)	2475–2700	Nepal (Golovatch 1990a, Golovatch and Martens 1996)
122. *A. cylindricus* (Carl, 1935)	1650–2850	Nepal & India, Darjeeling Distr. (Carl 1935, Golovatch 1984, Golovatch and Martens 1996, Golovatch and Wesener 2016)
123. *A. elongissimus* (Golovatch, 1984)	1000	India, Darjeeling Distr. (Golovatch 1984, Golovatch and Martens 1996, Golovatch and Wesener 2016)
124. *A. magnus* Golovatch, 2015	2700	Nepal (Golovatch 2015a)
125. *A. martensi* (Golovatch, 1990)	2250–3600	Nepal (Golovatch 1990a, 2014b, 2016a, Golovatch and Martens 1996)
126. *A. schawalleri* (Golovatch, 1990)	2050–2150	Nepal (Golovatch 1990a, Golovatch and Martens 1996)
127. *A. similis* (Golovatch, 1990)	2300–3000	Nepal (Golovatch 1990a, Golovatch and Martens 1996)
128. *A. spinosus* Golovatch, 2016	2500	Nepal (Golovatch 2016a)
129. *A. subcylindricus* (Carl, 1932)	?	S India & Nepal (Nguyen 2010^4^, Golovatch and Wesener 2016)
Genus *Beronodesmoides* Golovatch, 2015	1650–4250	
130. *Beronodesmoides anteriporus* Golovatch, 2015	1650–3350	Nepal (Golovatch 2015a, 2016c)
131. *B. bifidus* Golovatch, 2015	3100	Nepal (Golovatch 2015a)
132. *B. lobatus* Golovatch, 2015	4000–4250	Nepal (Golovatch 2015a, Golovatch et al. 2016)
133. *B. longifemoratus* Golovatch, 2015	2700–2800	Nepal (Golovatch 2016a)
134. *B. martensi* Golovatch, 2016	2700	Nepal (Golovatch 2016c)
135. *B. montigena* Golovatch, 2016	3550	Nepal (Golovatch 2016c)
136. *B. typicus* Golovatch, 2016	3400	Nepal (Golovatch 2016c)
Genus *Beronodesmus* Golovatch, 2014	1650–4500	
137. *Beronodesmus curtispinus* Golovatch, 2015	4500	Nepal (Golovatch 2015a)
138. *B. distospinosus* Golovatch, 2015	1650–3080	Nepal (Golovatch 2015a, 2016c)
139. *B. gorkhalis* Golovatch, 2015	3050–3600	Nepal (Golovatch 2015a, Golovatch et al. 2016)
140. *B. latispinosus* Golovatch, 2015	1900–3500	Nepal (Golovatch 2015a, 2016c, Golovatch et al. 2016)
141. *B. longispinus* Golovatch, 2015	2550–4270	Nepal (Golovatch 2015a, 2016c, Golovatch et al. 2016)
142. *B. martensi* Golovatch et al., 2016	2650	Nepal (Golovatch et al. 2016)
143. *B. minutus* Golovatch, 2015	3300–3500	Nepal (Golovatch 2015a)
144. *B. pallidus* Golovatch, 2014	3800–4100	Nepal (Golovatch 2014b)
145. *B. serratus* Golovatch et al., 2016	3300–3500	Nepal (Golovatch et al. 2016)
146. *B. simplex* Golovatch, 2016	2100	Nepal (Golovatch 2016c, Golovatch et al. 2016)
147. *B. sinuatospinus* Golovatch, 2015	2150–2250	Nepal (Golovatch 2015a, 2016c)
Genus *Delarthrum* Attems, 1936	600–4100	
148. *Delarthrum aberrans* (Golovatch, 1996)	1000–2600	Nepal (Golovatch 1996, 2014b, 2016a, Golovatch and Martens 1996)
149. *D. affine* (Golovatch, 1994)	1400	Nepal (Golovatch 1994a, 2014b, Golovatch and Martens 1996)
150. *D. alatum* (Golovatch, 1996)	1900–2100	Nepal (Golovatch 1996, 2014b, Golovatch and Martens 1996)
151. *D. andreevi* Golovatch, 2014	1800	Nepal (Golovatch 2014b)
152. *D. arunense* (Golovatch, 1994)	1850–2150	Nepal (Golovatch 1994a, 2014b, 2016a, Golovatch and Martens 1996)
153. *D. beroni* Golovatch, 2014	600–1000	Nepal (Golovatch 2014b)
154. *D. bifidum* (Golovatch, 1996)	2550–2650	Nepal (Golovatch 1996, 2014b, Golovatch and Martens 1996)
155. *D. chulingense* (Golovatch, 1994)	3000–3700	Nepal (Golovatch 1994a, 2014b, Golovatch and Martens 1996)
156. *D. communicans* (Golovatch, 1992)	2650	Nepal (Golovatch 1992, 2014b, Golovatch and Martens 1996)
157. *D. curtisoma* Golovatch, 2015	2050–2150	Nepal (Golovatch 2015a)
158. *D. curtum* Golovatch, 2014	600–1000	Nepal (Golovatch 2014b)
159. *D. densesetosum* Golovatch, 2015	2400	Nepal (Golovatch 2015a)
160. *D. elegans* (Golovatch, 1992)	1350	Nepal (Golovatch 1992, 2014b, Golovatch and Martens 1996)
161. *D. extremum* (Golovatch, 1996)	2450	Nepal (Golovatch 1996, 2014b, Golovatch and Martens 1996)
162. *D. facile* (Golovatch, 1996)	2200–2400	Nepal (Golovatch 1996, 2014b, Golovatch and Martens 1996)
163. *D. fechteri* (Golovatch, 1990)	2330–3150	Nepal (Golovatch 1990a, 2014b, Golovatch and Martens 1996)
164. *D. foveatum* (Golovatch, 1996)	1800–2000	Nepal (Golovatch 1992, 2014b, Golovatch and Martens 1996)
165. *D. furcatum* (Golovatch, 1996)	600–2000	Nepal (Golovatch 1996, 2014b, 2016c, Golovatch and Martens 1996)
166. *D. gracile* Golovatch, 2015	1750	Nepal (Golovatch 2015a)
167. *D. granulosum* (Golovatch, 1994)	2000	Nepal (Golovatch 1994a, 2014b, 2016c, Golovatch and Martens 1996)
168. *D. heterotergale* Golovatch, 2014	600–1000	Nepal (Golovatch 2014)
169. *D. hingstoni* (Carl, 1935)	3400	China, Tibet (Carl 1935, Golovatch and Martens 1996)
170. *D. hirsutum* (Golovatch, 1994)	2400–4100	Nepal (Golovatch 1994a, 2014b, 2015a, Golovatch and Martens 1996)
171. *D. intermedium* (Golovatch, 1994)	1000–1100	Nepal (Golovatch 1994a, 2014b, Golovatch and Martens 1996)
172. *D. invocatum* (Golovatch, 1996)	2600–2800	Nepal (Golovatch 1996, 2014b, Golovatch and Martens 1996)
173. *D. kuznetsovi* (Golovatch, 1994)	3000	Nepal (Golovatch 1994a, 2014b, Golovatch and Martens 1996)
174. *D. longisetum* (Golovatch, 1994)	1400–1600	Nepal (Golovatch 1994a, 2014b, 2016c, Golovatch and Martens 1996)
175. *D. longispinum* (Golovatch, 1996)	2150–2250	Nepal (Golovatch 1996, 2016c, Golovatch and Martens 1996)
176. *D. modestum* (Golovatch, 1996)	3450–3600	Nepal (Golovatch 1996, 2014b, Golovatch and Martens 1996)
177. *D. nyakense* (Golovatch, 1992)	2270–2400	Nepal (Golovatch 1992, 2014b, Golovatch and Martens 1996)
178. *D. obscurum* Attems, 1936	ca 2800	N Pakistan, Punjab (Attems 1936, Golovatch and Wesener 2016)
179. *D. philosophicum* (Golovatch, 1994)	1650–2450	Nepal (Golovatch 1994a, 2014b, Golovatch and Martens 1996)
180. *D. planifemur* Golovatch, 2015	2200	Nepal (Golovatch 2015a)
181. *D. prolixum* (Golovatch, 1996)	2550–2650	Nepal (Golovatch 1996, 2014b, Golovatch and Martens 1996)
182. *D. pumilum* (Attems, 1944)	?	India, Uttar Pradesh (Attems 1944, Golovatch and Wesener 2016)
183. *D. quadridentatum* Golovatch, 2016	2600–2800	Nepal (Golovatch 2016b)
184. *D. schawalleri* (Golovatch, 1992)	1000–2150	Nepal (Golovatch 1992, 1994a, 2014, Golovatch and Martens 1996)
185. *D. setosum* Golovatch, 2014	?	Nepal (Golovatch 2014b)
186. *D. silvestre* (Golovatch, 1994)	2000–3400	Nepal (Golovatch 1994a, 2014b, 2016a, Golovatch and Martens 1996)
187. *D. simile* (Golovatch, 1992)	2300–2700	Nepal (Golovatch 1992, 2014b, Golovatch and Martens 1996
188. *D. simplex* (Golovatch, 1996)	1650	Nepal (Golovatch 1996, 2014b, Golovatch and Martens 1996)
189. *D. simulans* (Carl, 1935)	3700	Nepal & China, Tibet (Carl 1935, Golovatch and Martens 1996)
190. *D. spectabile* (Golovatch, 1994)	2650	Nepal (Golovatch 1994a, 2014b, 2016c, Golovatch and Martens 1996)
191. *D. spiniger* (Attems, 1936)	1000–2200	India, West Bengal & Darjeeling Distr. (Attems 1936, Golovatch 1984, Golovatch and Wesener 2016)
192. *D. spinigerum* (Golovatch, 1992)	600–1400	Nepal (Golovatch 1992, 2014b)
193. *D. splendens* (Golovatch, 1992)	1650–2150	Nepal (Golovatch 1992, 1994a, 2014, Golovatch and Martens 1996)
194. *D. subalatum* (Golovatch, 1996)	2600–2800	Nepal (Golovatch 1996, 2014b, Golovatch and Martens 1996)
195. *D. subsimulans* (Golovatch, 1996)	3100–3300	Nepal (Golovatch 1996, 2014b, Golovatch and Martens 1996)
196. *D. tenuitergale* Golovatch, 2014	3250	Nepal (Golovatch 2014)
197. *D. tergale* (Golovatch, 1994)	2650	Nepal (Golovatch 1994a, 2014b, Golovatch and Martens 1996)
198. *D. tuberculatum* (Golovatch, 1994)	3000–3300	Nepal (Golovatch 1992, 1994a, 2014b, Golovatch and Martens 1996)
199. *D. typicum* Golovatch, 2014	3100	Nepal (Golovatch 2014)
200. *D. uncum* (Golovatch, 1996)	2100–3420	Nepal (Golovatch 1996, 2014, 2015a, Golovatch and Martens 1996)
201. *D. unicolor* (Attems, 1936)	1200–1700	India, Assam & Darjeeling Distr. (Attems 1936, Golovatch and Wesener 2016)
Genus *Hirtodrepanum* Golovatch, 1994		
202. *Hirtodrepanum latigonopum* Golovatch, 1994	2100–2600	Nepal (Golovatch 1994b, 2015a, Golovatch et al. 2016)
Genus *Kaschmiriosoma* Schubart, 1935	1000–3300	
203. *Kaschmiriosoma contortipes* Schubart, 1935	2000–3300	N Pakistan & India, Jammu & Kashmir (Schubart 1935, Silvestri 1936, Golovatch 1983, Golovatch and Martens 1996, Jeekel 2003, Shelley 2014)
204. *K. nulla* (Attems, 1936)	1000	India, Himachal Pradesh (Attems 1936, Golovatch and Martens 1996, Jeekel 2003, Golovatch and Wesener 2016)
205. *K. pleuropterum* (Attems, 1936)	2800	N Pakistan, Punjab (Attems 1936, Golovatch and Martens 1996, Jeekel 2003, Golovatch and Wesener 2016)
Genus *Kronopolites* Attems, 1914		
206. *Kronopolites coriaceus* Golovatch, 2015	2000	Nepal (Golovatch 2015a)
207. *K. occidentalis* Golovatch, 1983	1500	India, Jammu & Kashmir (Golovatch 1983, Golovatch and Martens 1996, Golovatch and Wesener 2016)
Genus *Orthomorpha* Bollman, 1893		
208. *Orthomorpha coarctata* (de Saussure, 1860)	600–650	Nepal & India, pantropical anthropochore (Golovatch and Martens 1996, Golovatch and Wesener 2016)
209. “*O.” almorensis* Turk, 1947	1600	India, Almora (Turk 1947, Golovatch and Martens 1996, Golovatch and Wesener 2016)
Genus *Oxidus* Cook, 1911		
210. *Oxidus gracilis* (C.L. Koch, 1847)	570–1200	Nepal & India, subcosmopolitan anthropochore (Golovatch and Martens 1996, Golovatch and Wesener 2016)
Genus *Pocockina* Jeekel, 1965		
211. *Pocockina schawalleri* Golovatch, 2016	150	Nepal (Golovatch 2016a)
Genus *Streptogonopus* Attems, 1914		
212. *Streptogonopus phipsoni* (Pocock, 1892)	≤2700	Pakistan, Bangladesh, Nepal & India, West Bengal (Golovatch 2015a, Golovatch and Wesener 2016)
Genus *Substrongylosoma* Golovatch, 1984	1000–2200	
213. *Substrongylosoma bifurcatum* Golovatch, 2016	2000	Nepal (Golovatch 2016a)
214. *S. distinctum* Golovatch, 1984	1200–1500	India, Darjeeling Distr. (Golovatch 1984, Golovatch and Martens 1996, Golovatch and Wesener 2016)
215. *S. falcatum* Golovatch, 1984	1000–1400	India, Darjeeling Distr. (Golovatch 1984, Golovatch and Martens 1996, Golovatch and Wesener 2016)
216. *S. exiguum* Golovatch, 2016	1900	Nepal (Golovatch 2016a)
217. *S. montigena* (Carl, 1935)	1200–2200	India, Darjeeling Distr. (Carl 1935, Golovatch 1984, Golovatch and Martens 1996, Golovatch and Wesener 2016)
218. *S. schawalleri* Golovatch, 1993	1620–2000	Nepal (Golovatch 1993, Golovatch and Martens 1996)
Genus *Sundanina* Attems, 1914		
219. “*Sundanina” septentrionalis* Turk, 1947	ca 1700	India, Almora (Turk 1947, Golovatch and Wesener 2016)
Genus *Topalosoma* Golovatch, 1984	900	
220. *Topalosoma setiferum* Golovatch, 1984	900	India, Darjeeling Distr. (Golovatch 1984, Golovatch and Martens 1996, Golovatch and Wesener 2016)
Genus *Trogodesmus* Pocock, 1895		
221. *Trogodesmus uncinatus* (Attems, 1936)	?	India, Assam (Attems 1936, Golovatch and Wesener 2016^5^)
Genus *Touranella* Attems, 1937	2300–2800	
222. *Touranella himalayaensis* Golovatch, 1994	2300–2700	Nepal (Golovatch 1994b, Golovatch and Martens 1996)
223. *T. pilosa* Golovatch, 2016	2600–2800	Nepal (Golovatch 2016b)
**Family Polydesmidae**	350–4250	
Genus *Bhutanodesmus* Golovatch, 1988		
224. *Bhutanodesmus velatus* Golovatch, 1988	350–450	Bhutan (Golovatch 1988, Golovatch and Martens 1996)
Genus *Epanerchodus* Attems, 1901	2300–4250	
225. *Epanerchodus buddis* (Golovatch, 1986)	3300–3400	Nepal (Golovatch 1986, Golovatch and Martens 1996, Golovatch et al. 2011)
226. *E. occultus* (Golovatch, 1986)	2300–2800	Nepal (Golovatch 1986, Golovatch and Martens 1996, Golovatch et al. 2011)
227. *E. sacer* (Golovatch, 1987)	3300–3400	Nepal (Golovatch 1987a, Golovatch and Martens 1996, Golovatch et al. 2011)
228. *E. theocraticus* (Golovatch, 1990)	2600–2800	Nepal (Golovatch 1990b, Golovatch and Martens 1996, Golovatch et al. 2011)
229. *E. theosophicus* (Golovatch, 1986)	3200	Nepal (Golovatch 1986, Golovatch et al. 2011, Golovatch and Martens 1996)
230. *Epanerchodus* sp.	3450–4250	Nepal & Bhutan (Golovatch and Martens 1996)
Genus *Glenniea* Turk, 1945	350–2800	
231. *Glenniea bhotiaensis* Golovatch, 1988	350–450	Bhutan (Golovatch 1988a, Golovatch and Martens 1996)
232. *G. indica* Turk, 1945	2800	India, Himachal Pradesh (Turk 1945a, 1945b, Golovatch 1988a, Golovatch and Martens 1996, Golovatch and Wesener 2016)
233. *G. martensi* (Golovatch, 1987)	1200	Nepal (Golovatch 1987b, 1988a, Golovatch and Martens 1996)
234. *G. minuscula* Golovatch, 1988	1900–2300	Bhutan (Golovatch 1988a, Golovatch and Martens 1996)
235. *G. perarmata* Golovatch, 1988	1680	Bhutan (Golovatch 1988a, Golovatch and Martens 1996)
Genus *Himalodesmus* Golovatch, 1986	1000–3400	
236. *Himalodesmus audax* Golovatch, 1986	2650	Nepal (Golovatch 1986, Golovatch and Martens 1996)
237. *H. benefactor* Golovatch, 1987	2600–3400	Nepal (Golovatch 1987a, Golovatch and Martens 1996)
238. *H. faustus* Golovatch, 1987	1000–1750	Nepal (Golovatch 1987a, Golovatch and Martens 1996)
239. *H. parvus* Golovatch, 1987	2200	Nepal (Golovatch 1987a, Golovatch and Martens 1996)
240. *H. prosperus* Golovatch, 1990	2600–2800	Nepal (Golovatch 1990b, Golovatch and Martens 1996)
241. *H. pulcher* Golovatch, 1987	2450	Nepal (Golovatch 1987a, Golovatch and Martens 1996)
242. *H. pygmaeus* Golovatch, 1986	3300–3400	Nepal (Golovatch 1986, Golovatch and Martens 1996)
243. *H. vigens* Golovatch, 1987	2150–2250	Nepal (Golovatch 1987a, Golovatch and Martens 1996)
Genus *Typhlopygmaeosoma* Turk, 1972		
244. *Typhlopygmaeosoma hazeltonae* Turk, 1972	1850	India, Himachal Pradesh (Turk 1972, Shear 1986, Golovatch 1988b, Golovatch et al. 2014)
**Family Trichopolydesmidae**	450–4500	
Genus *Assamodesmus* Manfredi, 1955		
245. *Assamodesmus lindbergi* Manfredi, 1954	?	India, Assam (Manfredi 1954, Golovatch 1988b, Golovatch and Martens 1996, Golovatch et al. 2014)
Genus *Hingstonia* Carl, 1935	2000–4500	
246. *Hingstonia beatae* Golovatch, 1990	2400–3500	Nepal (Golovatch 1990b, Golovatch and Martens 1996, Golovatch et al. 2014)
247. *H. dorjulana* Golovatch, 1988	2450–3100	Bhutan (Golovatch 1988a, Golovatch and Martens 1996, Golovatch et al. 2014)
248. *H. eremita* Carl, 1935	2000	Nepal (Carl 1935, Golovatch 1986, Golovatch and Martens 1996, Golovatch et al. 2014)
249. *H. falcata* Golovatch, 1986	2650	Nepal (Golovatch 1986, Golovatch and Martens 1996, Golovatch et al. 2014)
250. *H. fittkaui* Golovatch, 1990	3350–3450	Nepal (Golovatch 1990b, Golovatch and Martens 1996, Golovatch et al. 2014)
251. *H. gogonana* Golovatch, 1988	3650–4000	Bhutan (Golovatch 1988a, Golovatch and Martens 1996, Golovatch et al. 2014)
252. *H. pahakholana* Golovatch, 1990	2600–2800	Nepal (Golovatch 1990b, Golovatch and Martens 1996, Golovatch et al. 2014)
253. *H. pelelana* Golovatch, 1988	3300–3400	Bhutan (Golovatch 1988a, Golovatch and Martens 1996, Golovatch et al. 2014)
254. *H. perarmata* Golovatch, 1986	3150	Nepal (Golovatch 1986, Golovatch and Martens 1996, Golovatch et al. 2014)
255. *H. serrata* Golovatch, 1987	3400–3600	Nepal (Golovatch 1987a, Golovatch and Martens 1996, Golovatch et al. 2014)
256. *H. sympatrica* Golovatch, 1990	3550–3650	Nepal (Golovatch 1990b, Golovatch and Martens 1996, Golovatch et al. 2014)
257. *H. variata* Golovatch, 1987	2600–4500	Nepal (Golovatch 1987a, 1990b, Golovatch and Martens 1996, Golovatch et al. 2014)
258. *H. yeti* Golovatch, 1988	1600–2600	Bhutan (Golovatch 1988a, Golovatch et al. 2014)
259. *Hingstonia* sp.	2200–3900	Nepal (Golovatch and Martens 1996)
Genus *Magidesmus* Golovatch, 1988	3100–3400	
260. *Magidesmus affinis* Golovatch, 1988	3300–3400	Bhutan (Golovatch 1988a, Golovatch et al. 2014)
261. *M. bhutanensis* Golovatch, 1988	3100	Bhutan (Golovatch 1988a, Golovatch et al. 2014)
Genus *Pseudosphaeroparia* Carl, 1932		
262. *Pseudosphaeroparia cavernicola* Turk, 1945	2800	India, Uttar Pradesh (Turk 1945a, 1945b, Golovatch and Martens 1996, Golovatch et al. 2014)
Genus *Sholaphilus* Carl, 1932	1100–2200	
263. *Sholaphilus asceticus* Golovatch, 1986	1300–1650	Nepal (Golovatch 1986, Golovatch and Martens 1996, Golovatch et al. 2014)
264. *S. dalai* Golovatch, 1986	2400	Nepal (Golovatch 1986, Golovatch and Martens 1996, Golovatch et al. 2014)
265. *S. gompa* Golovatch, 1990	2000–2100	Nepal (Golovatch 1990b, Golovatch and Martens 1996, Golovatch et al. 2014)
266. *S. lama* Golovatch, 1986	1800–2000	Nepal (Golovatch 1986, Golovatch and Martens 1996, Golovatch et al. 2014)
267. *S. martensi* Golovatch, 1986	1100–1850	Nepal (Golovatch 1986, Golovatch and Martens 1996, Golovatch et al. 2014)
268. *S. monachus* Golovatch, 1990	2050–2150	Nepal (Golovatch 1990b, Golovatch and Martens 1996, Golovatch et al. 2014)
Genus *Topalodesmus* Golovatch, 1988		
269. *Topalodesmus communis* Golovatch, 1988	2000–2200	India, Darjeeling Distr. (Golovatch 1988b, Golovatch and Martens 1996, Golovatch et al. 2014)
**Family Pyrgodesmidae**	450–1200	
270–275? Several genera and species (including at least 2 species of *Cryptocorypha* Attems, 1907)	450–1200	Nepal (Golovatch and Martens 1996)

^1^ A large, still unidentified species of *Monographis* is available from E Nepal, taken at 2400 m a.s.l. (M. Short, in litt.). Because the genus is feminine in gender, the species (adjective) must be named “*mira*”.^2^ A still unidentified species of *Unixenus* is available from E Nepal, taken at 3600–3900 m a.s.l. (M. Short, in litt.).^3^ The nominal subspecies is known only from S India (Silvestri 1923).^4^ The first record by Nguyen (2010) of this south Indian species from Nepal seems to be erroneous, based on no evidence whatsoever.^5^ Nguyen and Sierwald (2013) erroneously stated this species as deriving from Myanmar.

## Results

### Species of Diplopoda

Species concepts are only little addressed in diplopod taxonomy. To think about species limits and species definitions is not at all trivial; in nearly every case, a morphological species concept is used with the background idea that these entities, defined by external characters, fit well to the Biological Species Concept. In practical alpha-taxonomy it circumscribes reproductively isolated groups of specimens. Diplopod taxonomists largely base their identifications on adult male samples. Differences in male genitalic structure usually provide the basic characters that allow us to safely determine millipede species. In most cases this raises no problems. In the Himalayas, however, we have to tackle with numerous populations in a wide array of forest habitats found at various altitudes and in remote and secluded valleys. Hardly surprisingly, the Himalayas do support quite a number of examples of species swarms among Diplopoda as well. Species delimitation may then cause problems like those described by Martens (1978) for the polymorphous biantid harvestman *Biantes pernepalicus* Martens, 1978. Such situations also resemble the few known cases of insular species swarms in millipedes of Macaronesia (e.g. Enghoff 1992).

**Figure 3.**
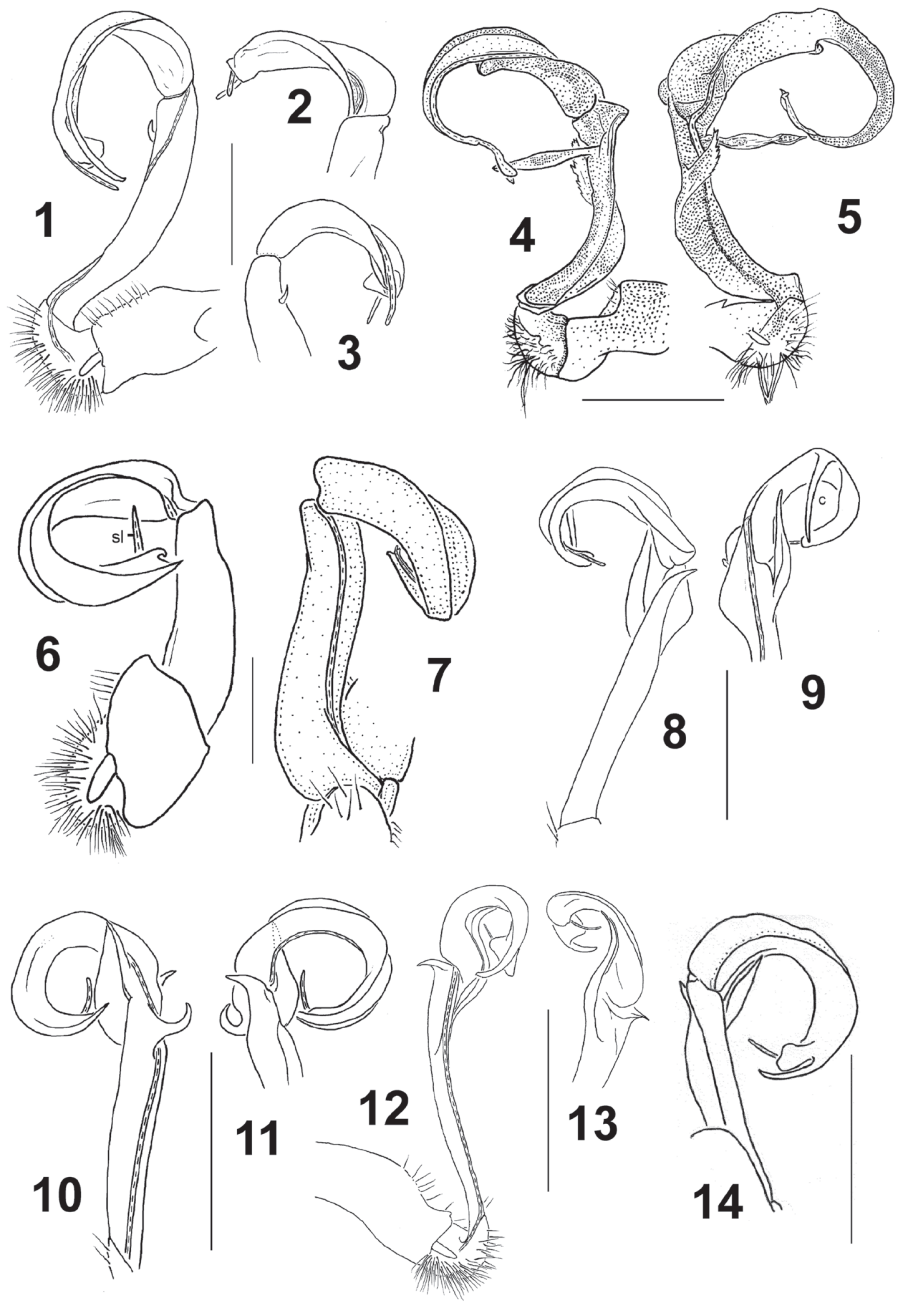
Gonopodal structural variations between several species of *Beronodesmus*: *B. martensi* Golovatch et al., 2016 (**1–3**), *B. serratus* Golovatch et al., 2016 (**4, 5**), *B. simplex* Golovatch, 2016 (**6, 7**), *B. distospinosus* Golovatch, 2015 (**8, 9**), *B. latispinosus* Golovatch, 2015 (**10, 11**), B. *sinuatospinus* Golovatch, 2015 (**12, 13**) and *B. gorkhalis* Golovatch, 2015 (**14**). Scale bars: 1.0 mm (**4–5, 14**), 0.5 mm (**1–3, 8–13**) or 0.4 mm (**6, 7**). After Golovatch (2015a, 2016c) and Golovatch et al. (2016).

The following examples can be given and easily added to the roster of similar observations that Martens (2015) made or compiled for Himalayan mammals, birds, arachnids, insects and several other animal groups. Such a distribution pattern can be termed fanned (see below) and is also found in the endemic Himalayan diplopod genera *Beronodesmus* and *Beronodesmoides* containing 11 and seven species, respectively (Golovatch 2016c, Golovatch et al. 2016). The main species-specific characters are in minor details of gonopodal structure, in particular, the shapes of the various outgrowths (Fig. 3). Vicariance speciation must have taken place *in situ*, with several of the congeners forming pairs or trios that can occur sympatrically or even syntopically and thus implying a series of secondary dispersal events.

### Zoogeographical patterns and origins

**Ecology and dispersal abilities**

The vast majority of Himalayan diplopod species are highly localized in distribution, both geographical and altitudinal. There are only few relatively widespread species like *Zephronia manca*, *Siphonorhinus cingulatus* (both recorded from Vietnam & Darjeeling District, India) or *Trichopeltis watsoni* (Bangladesh, Myanmar, Bhutan and India, Darjeeling Distr., West Bengal, Assam & near Kolkata). Despite extended collection acitivities over most parts of Nepal during several decades, most millipedes in the Himalayas remain known from only a single or very few localities. This particularly concerns the best-explored fauna of Nepal, Central Himalayas (ca 160 spp.), including species of the dominant family Paradoxosomatidae (82 spp., or >50%).

**Figure 4.**
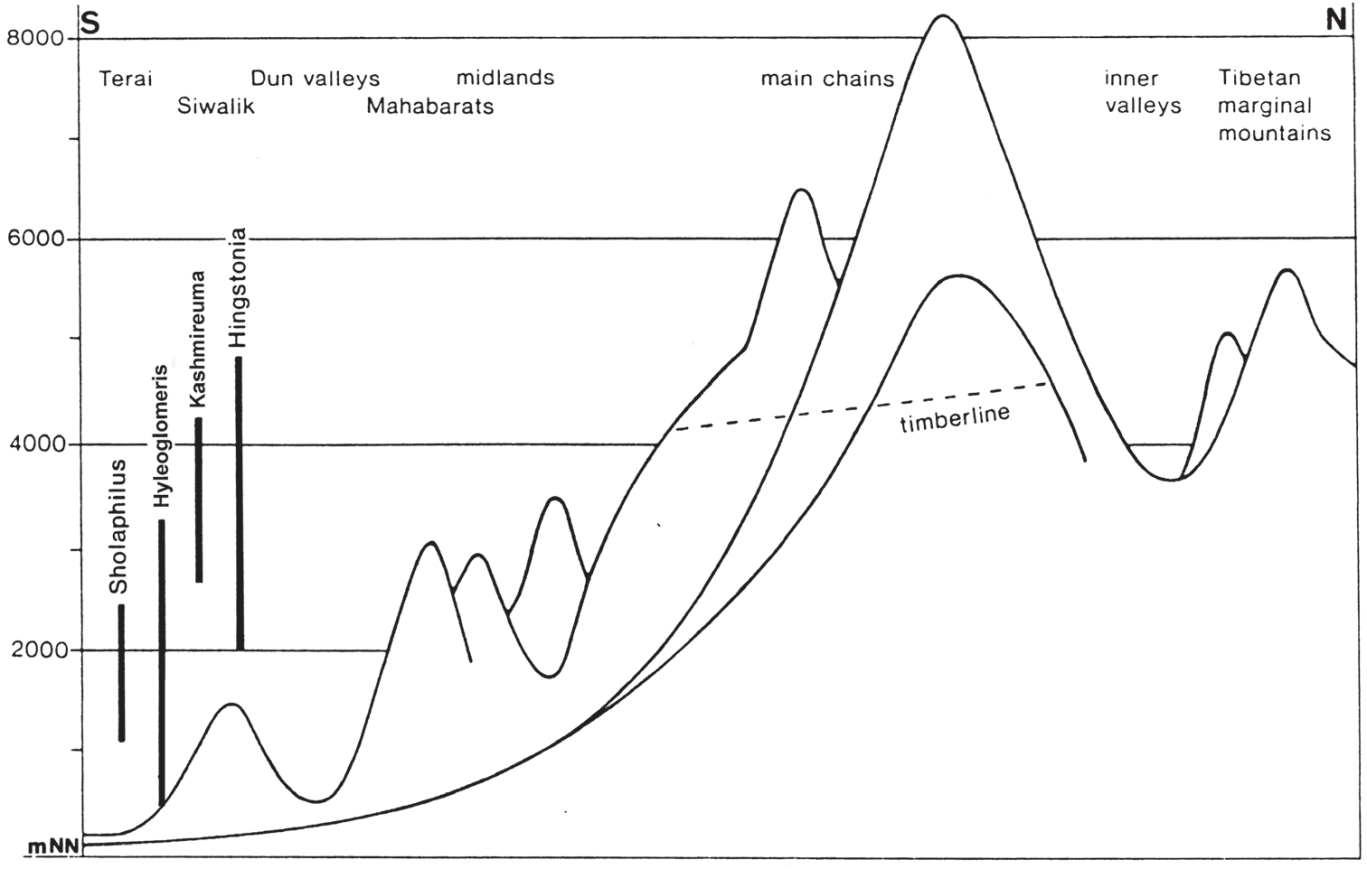
Vertical distribution of several genera of Diplopoda in the Himalayas (modified, after Golovatch and Martens 1996).

In contrast, most genera occur through a range of altitudes (Fig. 4), but are more or less restricted to forest habitats. The alpine (= nival) zone of the Central Himalayas which lies above closed forests appears to only marginally be populated by Diplopoda (Table 1, Figs 2, 4), the bulk of the fauna being confined to the tropical and subtropical forest belts. The highest records belong to *Nepalozonium trimaculatum* and *Nepalmatoiulus ivanloebli*, both found at 4800 m a.s.l. This pattern conforms to general knowledge that millipedes are basically a class of forest-dwelling terrestrial arthropods both trophecologically and historically largely associated with woodlands and ranging from nemoral (= broadleaved forest) and coniferous forest in temperate regions in the north to rainforest tropical areas in the south (Golovatch 1997a, 1997b). Such a background is accepted and it serves as the basis for faunogenetic reconstructions using phyto- and palaeogeographical evidence.

According to Martens (1993, 2015), broad vertical distribution belts appear to be exceptional in animals at least in the Central Himalayas, in contrast to fanned ones which are especially characteristic of species-rich groups, both vertebrates and invertebrates. Yet the vertical width of a distributional belt varies very considerably between taxa. Diplopods in their natural habitats seem to be particularly restricted to their forest habitats, with little capacity for enlarging their areas, both vertically and horizontally. There is hardly any other larger land arthropod group that shows a similarly strong specialization and relation to a habitat. In many cases, congeners occupy only limited vertical belts and such patterns are the result of multi-layered speciation processes that occurred in secluded valleys and mountain massifs. There are obvious altitudinal adaptations resulting from the interaction between Pliocene/Pleistocene climate oscillations and speciation processes. Whilst species of different clades occupy narrow vertical belts, close congeners, i.e. sister species, are mostly horizontally allopatric because of their allopatric vicariance speciation. Sympatry or syntopy are quite common among congeners (see above for *Beronodesmus*, Fig. 3), this alone implying a series of secondary dispersal events. On the other hand, all 13 species of *Nepalmatoiulus* known from Nepal and Bhutan are nearly exclusively allopatric in distribution (Enghoff 1987).

Narrow belts seem to be rare, when each individual species has been recorded from a single or very few localities, but even the whole species set combined remains restricted to a narrow altitudinal range. Much more common, rather usual are the situations when the vertical and horizontal distribution of a species is highly restricted, but that of the corresponding species-group or genus is very considerable (Table 1, Fig. 4).

Among the Diplopoda of the Himalayas, most if not all of the rather to highly species-rich genera show fanned vertical distribution patterns. Such are all genera at least in the orders Sphaerotheriida, Glomerida, Chordeumatida, Julida and Spirostreptida, as well as most in the order Polydesmida. Their origins seem to be very different, but profound *in situ* speciation is their general characteristic. No evident narrow belts seem to be distinguishable in the combined vertical distribution of millipede congeners in the Himalayas (Table 1, Figs 2, 4). Even within Nepal, a gradual east-west decline in diplopod diversity is clearly observed towards the country’s central regions, apparently following the climate aridity gradient and increased absence of humid forest.

As noted above, zoogeographically the Himalayas are traditionally viewed as a vast, yet clear-cut transitional zone between the Palaearctic and Oriental (= Indo-Malayan) realms. Martens (2015, p. 212) writes that “The renowned species diversity of the Himalayan fauna results from the area’s position between tropical SE Asia to the south and southeast, temperate High Asia to the north and dry Central Asia to the northwest, in addition to its proximity to endemic-rich SW China, which harbours many Tertiary relicts. Elements from all these areas contributed to and influenced the present faunal composition, creating one of the world’s 25 biodiversity hotspots, Indo-Burma and the adjacent South Central China.” He discriminated a predominantly immigrant fauna with five main sources: SW Chinese Himalayan from the northeastern Himalayan Arc; Indochinese Himalayan from the southeast; tropical Indian from the south; central Asian from the north via Tibet; and W Asian Himalayan from mountain ranges westwards to the Mediterranean. This fauna is mainly composed of species with good dispersal abilities such as bats, birds, butterflies, dragonflies and many other groups. Since millipedes are poor dispersers and only relatively few species are anthropochores, we refrain from enlisting any as belonging to this category, even though a few widespread Oriental species do reach the Himalayas from the east and/or southeast (e.g. the above *Zephronia manca*, *Siphonorhinus cingulatus*, *Streptogonopus phipsoni*, *Gonoplectus malayus malayus* or *Trichopeltis watsoni*). Human introductions cannot be excluded in such cases, these being especially apparent as regards the few unquestioned anthropochores like *Trigoniulus corallinus*, *Orthomorpha coarctata* or *Oxidus gracilis*.

In addition, a Sino-Himalayan fauna (or even a Sino-Himalayan region) is distinguished, which is characterized by remarkable diversity, partially of Indo-Malayan origins and partially Palaearctic in nature, but with marked peculiarities. Holt et al. (2012), based on the modern distributions and phylogenies of amphibians, birds and non-marine mammals, defined 20 distinct zoogeographic regions grouped into 11 realms, including a Sino-Japanese realm which appears to show closer phylogenetic affinities to the Palaearctic than to the Oriental realm. It includes Japan, Tibet and nearly all of China. Eventually, that paper represents one of the most consistent, but no less unsuccessful attempts at uncritically combining the landscape-typological (= zonal) and faunogenetic approaches to biogeography which, however, must be clearly separated at least as regards the biotas of older biomes (e.g. Chernov 1975, Golovatch 2015b). To reiterate Chernov (1975) briefly, from the “viewpoint” of a biome or more local landscape it only matters whether the constituent species properly function as its biotic elements, regardless of their origins. In contrast, from a faunogenetic point of view, the more ancient the biome or landscape, the more ancient its biota and the more complex its history. Thus both approaches must be clearly distinguished, especially as regards the relatively younger zonal biomes like tundra or taiga (= boreal forest) from the particularly ancient, rather regional than zonal, subtropical and tropical ones.

**Figure 5.**
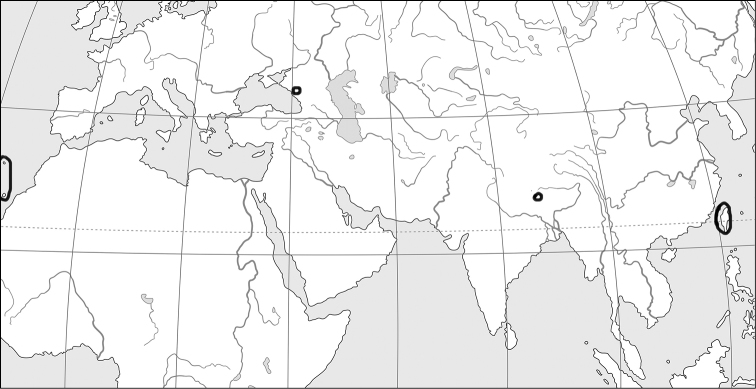
Distribution of the genus *Hirudicyptus* (Siphonocryptidae, Siphonocryptida). After Golovatch et al. (2015).

In terms of its faunal composition, the Sino-Himalayan region represents a mixed zone of elements derived from both the Palaearctic and Oriental realms, but it includes moreover a wealth of endemics with surprisingly small and often relict distributions (Martens 2015). It is within this category that many of the Himalayan Diplopoda seem best to place. Perhaps the most conspicuous example of such a pattern is represented by the definitely relict order Siphonocryptida which globally contains only two genera and seven species. Thus, the genus *Siphonocryptus* Pocock, 1894, comprises three species: one in Sumatra, Indonesia, the other two in continental Western Malaysia. In contrast, the distribution pattern of *Hirudicryptus* Enghoff & Golovatch, 1995 is trans-Palaearctic (Fig. 5). The type species occurs only on Madeira and the Canaries, where it is largely confined to the relict, subtropical, laurisilva biome. One species each is endemic to Taiwan, to Nepal and to the NW Caucasus (Golovatch et al. 2015, Zuev 2017). It may well be that the distribution pattern under consideration dates back at least to the Oligocene times of the so-called “Warm Earth” to have highly probable explanations rooted in palaeobotanical evidence. These imply a gradual shrinkage and disruption ever since of the previously dominating and continuous subtropical biome (Golovatch 1997a, 1997b, Zherikhin 2003). Being so vastly disjunct, the present-day distribution of Siphonocryptida is best accounted for by extinction events (Shelley and Golovatch 2011).

**Genus- and species-level relations**

Further possible examples of the Sino-Himalayan pattern seem to be represented by *Glenniea* (Polydesmidae, a largely Holarctic family), with five species from the Himalayas of Nepal and Bhutan, and three in S China (Golovatch 2015b), *Nepalella* (Megalotylidae, the genus being the westernmost in that temperate to tropical Asian family), with 23 species or subspecies from Nepal, S China and SE Asia (Minelli 2015, Table 1), *Hirtodrepanum* (Paradoxosomatidae, a subcosmopolitan family absent only from N America) with one species each in Nepal and S China (Golovatch 2014a), and *Martensodesmus* (Opisotretidae, the genus being the westernmost in that Indo-Australian family), with at least five species from the Himalayas of Nepal and Bhutan, two in S China, and one in S Vietnam (Golovatch et al. 2013). The latter example, however, may likewise illustrate the predominating zoogeographical connections of the Himalayan fauna with the Indo-Malayan one. Such are also the genera *Cryptocorypha* (Pyrgodesmidae), with 14 described species from S India, Myanmar, S China and Australasia, eastwards to Vanuatu, Melanesia (Golovatch and VandenSpiegel 2015, Golovatch and Wesener 2016), *Zephronia* (Sphaerotheriida), with 38 species or subspecies from Nepal, NE India, Myanmar, Thailand, Cambodia, Vietnam, Western (= mainland) Malaysia and Hong Kong (Wesener 2016), *Siphonorhinus* (Siphonophorida), with eight species from Indonesia, Vietnam, Cambodia, Laos, India and Madagascar (Minelli 2015) and *Pocockina* (Paradoxosomatidae), with three species from Nepal and Myanmar (Golovatch 2016b). The small genus *Trichopeltis* (Cryptodesmidae) includes nine described species, largely from Indochina, Sumatra, Indonesia, Myanmar and S China; only the much more widespread *T. watsoni* and *Gonoplectus malayus malayus* reach as far west as the Himalayas of India (Table 1). Similarly, amongst the Paradoxosomatidae the genus *Trogodesmus* contains four species in Myanmar and one more in Assam, India (Nguyen and Sierwald 2013); *Touranella* harbours two species from Nepal and further four from Vietnam (Golovatch 2016b); *Kronopolites* has one species in Jammu & Kashmir, India, one in Thailand, one in Laos, and another nine in China, including one in Taiwan (Likhitrakarn et al. 2015, Golovatch 2015a); *Anoplodesmus* is a large genus which, regardless of a few pantropical anthropochores, comprises approximately 35 species in S (India and Sri Lanka), E (Taiwan) and SE Asia (eastwards up to Sumatra, Indonesia), including nine congeners confined to the Himalayas (Golovatch 2016a, Table 1); *Streptogonopus* contains not only *S. phipsoni*, a species quite widespread in the Himalayas and certain adjacent countries (Table 1), but also two more in India, and one each in Eritrea, Thailand and Vietnam (Nguyen et al. 2016); *Delarthrum* is one of the most species-rich genera of Diplopoda (56 spp.), with most (55 spp.) of its diversity confined to the Himalayas of Pakistan, Nepal and India (Table 1), and only one outlier congener in S India (Golovatch and Wesener 2016, Golovatch 2016b). Much like *Delarthrum*, the genus *Sholaphilus* demonstrates faunal connections between the Himalayas (6 spp., Table 1) and S India (1 sp., Golovatch and Wesener 2016).

**Suprageneric relations**

At these taxonomic levels, the fully to largely tropical orders Sphaerotheriida, Siphonophorida and Spirostreptida, as well as most of Spirobolida, Platydesmida and Polydesmida (at least some Haplodesmidae, Cryptodesmidae, Paradoxosomatidae, Pyrgodesmidae and Trichopolydesmidae) seem to represent Indo-Malayan elements in the Himalayan millipede fauna. A siphonophoridan and a cryptodesmid species, both have been recorded as far north as N Pakistan (Golovatch 1991). However, at the species level the bulk of diversity is endemic and highly localized, both horizontally and vertically, clearly due to allopatric vicariance (cf. Golovatch and Martens 1996). There are several Himalayan endemic to subendemic diplopod genera, but not a single suprageneric taxon: *Kophosphaera* (Sphaerotheriida, seven species, Wesener 2016 & Table 1), *Koponenius* (Polydesmida, four species from Nepal, NW India and Myanmar (Golovatch and VandenSpiegel 2014, 2016), *Kashmireuma* (Chordeumatida), with three species from Nepal and N India (Table 1), *Himalodesmus* (Polydesmidae, Table 1), *Beronodesmoides, Beronodesmus*, *Substrongylosoma* (all Paradoxosomatidae, Table 1), *Magidesmus* (Trichopolydesmidae, Table 1), as well as the monotypic *Assamodesmus*, *Bhutanodesmus*, *Topalodesmus*, *Topalosoma* and *Typhlopygmaeosoma* (Table 1).

Faunal connections to the northwest and/or north are demonstrated by a few examples only. Even at the generic level, not all of them could unequivocally be treated as likely Palaearctic components in the Himalayan millipede fauna. Thus, the genus *Kaschmiriosoma* (Paradoxosomatidae) is composed of three species endemic to N Pakistan, and one to both N Pakistan and N India (Jeekel 2003). Such a pattern might seem to have been accounted for by an originally northwestwards dispersal. Even in the absence of a phylogenetic reconstruction, the gonopodal structure in *Kaschmiriosoma*, especially the particularly complex, strongly coiled and thus apomorphous solenophores as observed in the southernmost, Himalayan *K. contortipes* and *K. nulla*, may rather be evidence of a Palaearctic origin of the genus. Its deemed closest relatives within the tribe Sulciferini, also often showing particularly complex and strongly twisted gonopodal solenophores, are observed in the genera *Gonobelus* Attems, 1936, *Inversispina* Zhang, in Zhang et al. 1997 and a few others, all confined to southern China, occasionally including Taiwan (Jeekel 1980, Golovatch 2012, 2016b).

Ties to the north are much better pronounced, e.g., in the genera *Tianella* (Cleidogonidae), *Epanerchodus* (Polydesmidae), *Bollmania* (Caspiopetalidae) and *Anaulaciulus* (Julidae). *Tianella* has two described and a number on still undescribed species in Kyrgyzstan and Kazakhstan, Central Asia, as well as 11 named species in Nepal and a few undescribed ones from both Nepal and N India (Mauriès 1988, Read and Golovatch 1994, Table 1). *Epanerchodus* is a very large genus comprising 70+ species from Central and E Asia: Russian Far East, Korea, China, Taiwan and Japan, as well as several species from Nepal (Minelli 2015, Table 1). *Bollmania* is composed of eight described and a few undescribed species ranging from Turkmenistan, Iran, Uzbekistan, Tajikistan and Afghanistan in Central Asia to S China; one species is known from the Himalayas of Pakistan (Stoev et al. 2008, Table 1). *Anaulaciulus* is also a highly speciose Asian genus which contains nearly 50 species ranging from across the Himalayas, through China, to the Russian Far East, Korea, Japan and Taiwan (Korsós 2001, Table 1). It is partly sympatric with still another, similarly large, Asian genus *Nepalmatoiulus* (Julidae), which harbours 55 species also distributed across the Himalayas, but then extending more to the east and southeast (S China, Taiwan, S Ryukyus, Myanmar, Indochina and peninsular Malaysia) (Enghoff 1987, Korsós and Lazányi 2013, Table 1). In the Himalayas, many species from these genera are high-montane (Table 1), thus reinforcing their presumed Palaearctic origins. The occurrence of the sole known species of *Nepalozonium* (Polyzoniida) at 4700–4800 a.s.l., i.e. among the highest records in the entire class, coupled with the family Hirudisomatidae where it belongs being strictly Holarctic (Minelli 2015), is clearly evidence of its Palaearctic roots.

The pattern demonstrated by the very large genus *Hyleoglomeris* (Glomeridae) strongly resembles that of the family Siphonocryptidae (see above and Fig. 5), but in no way is it relictual. Indeed, its 100+ species range from the Balkans and Greek islands in the west, through Anatolia, the Caucasus, Central Asia and the Himalayas, to China, Korea, Japan, and Taiwan in the east, and through Indochina to the Philippines and Sulawesi, Indonesia in the southeast (Golovatch et al. 2006, Table 1). This picture actually reflects one of the fundamental patterns of historical biogeography as evidenced in the entire class Diplopoda (Shelley and Golovatch 2011). Generally, west-east trans-Himalayan faunal connections are traced in numerous millipede higher taxa: Glomerida, Julida, Chordeumatida, Callipodida, Siphonocryptida, Spirostreptida, Polydesmida etc. Southeast Asia is the only corner in the world where all 16 extant orders of Diplopoda are still to be found. In many cases, the Himalayas might have served as a paramount stepping stone and refugium in linking, much more in the past than at present, the faunas of SE Asia to those of Europe and W Asia. In the past, the Himalayas started rising and absorbing surrounding faunal elements often already present in the area. After having gained a certain height, the Himalayas functioned more as a trap, a “prison”, mountain ridges and deep valley systems hindering further faunal exchange, with the tremendous speciation process that came into action. Old migration routes both ways are thereby evident, although the influence of the Oriental fauna is by far greater. Since the uplift of the Himalayas started in the early Oligocene (about 27 Mya), the diplopod fauna of the region has gradually acquired its own, highly characteristic, very rich and diverse composition, multi-layered and very complex, with profound *in situ* radiations and vicariance events (cf. Golovatch and Martens 1996, Martens 2015). At least some of the oldest components are presently highly disjunct and clearly relict, as is the order Siphonocryptida (see above & Fig. 5). The most recent faunal layer is certainly represented by the few pantropical or subcosmopolitan introductions like *Trigoniulius corallinus, Orthomorpha coarctata* or *Oxidus gracilis*.

## Conclusions

Since the previous review of millipede chorology and faunogenesis in the Himalayas (Golovatch and Martens 1996), our knowledge of the Himalayan fauna has become considerably enriched (ca 200 vs >270 spp.) and often refined taxonomically. This is particularly true of the composition of the dominant family Paradoxosomatidae. However, the main results and trends remain unchanged.

**Figure 6.**
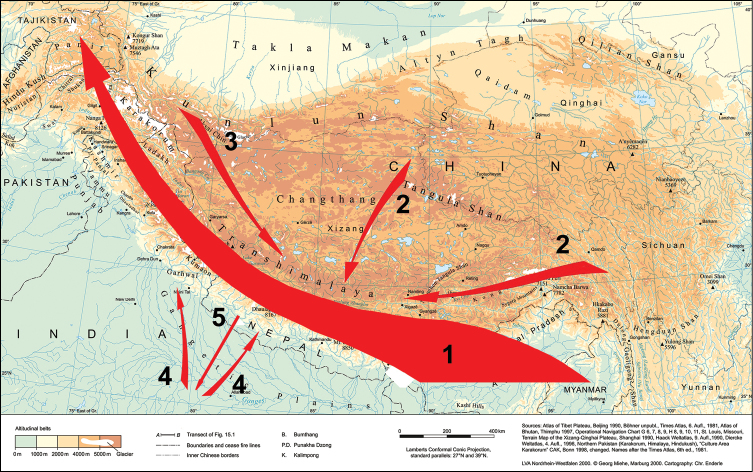
General schematic picture of the faunogenesis of Himalayan Diplopoda. Arrows reflect the main pathways of faunal migration or exchange, their thickness roughly corresponding to the degree of influence. The thickest arrow 1 clearly emphasizes the dominant roles the Indo-Malayan core fauna may have played in the present-day composition of the Himalayan fauna, its most ancient layers extending westwards to reach central and western Asia, as well as Europe (by default also northwards up to eastern Asia and even North America). The considerably less thick arrows 2 and 3 are to reflect the more subordinate roles the Sino-Himalayan and Palaearctic elements, respectively, could have played in the modern Himalayan fauna. Arrows 4 and, especially, 5 are even less thick and demonstrate the relatively minor faunal exchanges to be presumed between the Indian and Himalayan faunas.

The Himalayas support a highly rich, diverse, multi-layered, mostly endemic diplopod fauna. This is the result of mixing the ancient, apparently Tertiary and younger, Plio-Pleistocene elements of various origins, as well as the most recent anthropochore introductions. At the species and, partly, generic levels, the fauna is largely autochthonous and sylvicolous, formed through abounding *in situ* radiation and vicariance events, when overall the species from large genera and families tend to occupy a wide range of altitudes, but nearly each of the constituent species shows a distribution highly localized both horizontally and altitudinally, yet quite often with sympatry or even syntopy involved. The bulk of the fauna is Indo-Malayan in origin, with individual genera or families shared with those of SE Asia (mostly) and/or S India (few) (Fig. 6). Their constituent species tend to be lowland to mid-montane, but the general rule “(sub)tropical organisms for (sub)tropical environments only” fails very often.

The Palaearctic influence is modest (Fig. 6), but still can be traced in several genera and families. Collectively, their species tend to be high montane, but with numerous exceptions as well. The trans-Himalayan faunal connections at higher taxonomic levels, generic to ordinal, that link SE Asia to Europe are manifest. They show routes and directions of ancient dispersal both ways, but the one from SE Asia seems to have prevailed over the opposite one. Moreover, certain fragments or offshoots of such ancient, obviously Tertiary, opposite migrations more or less along the southern coasts of the receding Tethys Sea may have been left *en route* in S and SW China, as well as in N Pakistan and Central Asia.

One must also take into account that a number of presumably Himalayan species groups might have originated from the times when Tibet was still forest-covered and the Himalayan chain still in its infancies. According to Schmidt (2006), stem species invaded the raising Himalayas from the north where they developed to presently known species swarms. After the Himalayas and Tibetan Plateau had raised sufficiently high, Tibet became drier and the forests vanished including their fauna, the Himalayas becoming their exile. Schmidt coined the term “Tibeto-Tertiary element” of paleo-Tibetan origin with present Himalayan distributions (Schmidt 2006, Schmidt et al. 2012).

The particularly rich Himalayan diplopod fauna with its numerous small-ranging species confined to permanent forest sheds new light on a much disputed controversy among geographers, zoologists, taxonomists, climatologists and glacialogists (Kuhle 1982, 2015 and figure 4 therein). Was the central Himalayan chain, at least at certain sections of the Pleistocene, covered by a complete shield of ice? Taxonomists dealing with low-dispersal soil/litter-dwelling arthropods have a clear response. Any ice cover would have been detrimental to the local soil arthropod fauna and would have driven its larger part or entirely to extinction. Only a long and steady evolution under more or less constant, albeit slightly varying, conditions would have allowed the biota to develop gradually over long geological periods (Martens 2015). This scenario certainly applies to all Himalayan Diplopoda.

The above picture of faunal connections (Fig. 6) is consistent both with general wisdom (e.g. Martens 2015) and our previous analysis (Golovatch and Martens 1996), the salient aspects of Diplopoda, contrary to many other animal groups in the Himalayas, being their pronounced sylvicoly, extremely diverse and small-ranging species endemism, and mostly Oriental and/or Indian origins, while some of the rather ostensible influence of the Palaearctic may have come not only from the north and/or northwest, but also from the currently subtropical regions of S China. Reciprocal migrations from the Himalayan faunal knot as a major refugium and secondary diversification centre also seem quite plausible, but documenting such requires detailed phylogenies which unfortunately are still almost missing.
